# Distribution of serotypes and antibiotic resistance of invasive *Pseudomonas aeruginosa* in a multi-country collection

**DOI:** 10.1186/s12866-021-02427-4

**Published:** 2022-01-06

**Authors:** Shamima Nasrin, Nicolas Hegerle, Shaichi Sen, Joseph Nkeze, Sunil Sen, Jasnehta Permala-Booth, Myeongjin Choi, James Sinclair, Milagritos D. Tapia, J. Kristie Johnson, Samba O. Sow, Joshua T. Thaden, Vance G. Fowler, Karen A. Krogfelt, Annelie Brauner, Efthymia Protonotariou, Eirini Christaki, Yuichiro Shindo, Andrea L. Kwa, Sadia Shakoor, Ashika Singh-Moodley, Olga Perovic, Jan Jacobs, Octavie Lunguya, Raphael Simon, Alan S. Cross, Sharon M. Tennant

**Affiliations:** 1 Center for Vaccine Development and Global Health, University of Maryland School of Medicine, 685 W. Baltimore St. – HSF1 Room 480, Baltimore, MD 21201 USA; 2 Department of Medicine, University of Maryland School of Medicine, Baltimore, MD USA; 3 Department of Pediatrics, University of Maryland School of Medicine, Baltimore, MD USA; 4 Department of Pathology, University of Maryland School of Medicine, Baltimore, MD USA; 5 Centre pour le Développement des Vaccins, Mali, Bamako, Mali; 6 Division of Infectious Diseases, Duke University Medical Center, Durham, NC USA; 7 Division of Infectious Diseases and International Health, Department of Medicine, Duke University School of Medicine, Durham, NC USA; 8 Duke Clinical Research Institute, Durham, NC USA; 9 Statens Serum Institut, Copenhagen, Denmark; 10 Department of Natural Sciences and Environment, Roskilde University, Roskilde, Denmark; 11 Department of Microbiology, Tumor and Cell Biology, Division of Clinical Microbiology, Karolinska Institutet and Karolinska University Hospital, 17176 Stockholm, Sweden; 12 Department of Microbiology, AHEPA University Hospital, Thessaloniki, Greece; 13 Department of Medicine, AHEPA University Hospital, Thessaloniki, Greece; 14 University of Cyprus Medical School, Nicosia, Cyprus; 15 Department of Respiratory Medicine, Nagoya University Graduate School of Medicine, Nagoya, Japan; 16 Department of Pharmacy, Singapore General Hospital, Singapore, Singapore; 17 Emerging Infectious Diseases, Duke-National University of Singapore Medical School, Singapore, Singapore; 18 Department of Pharmacy, Faculty of Science, National University of Singapore, Singapore, Singapore; 19 Departments of Pathology and Pediatrics, Aga Khan University, Karachi, Pakistan; 20 National Institute for Communicable Diseases a Division of the National Health Laboratory Service, and School of Pathology, Faculty of Health Sciences, University of the Witwatersrand, Johannesburg, South Africa; 21 Department of Clinical Sciences, Institute of Tropical Medicine, Antwerp, Belgium; 22 Department of Microbiology, Immunology and Transplantation, KU Leuven, Leuven, Belgium; 23 Department of Clinical Microbiology, National Institute for Biomedical Research, Kinshasa, Democratic Republic of the Congo; 24 Department of Microbiology, University Hospital of Kinshasa, Kinshasa, Democratic Republic of the Congo

**Keywords:** Pseudomonas, Serotype, Flagellin, Multidrug resistance

## Abstract

**Background:**

*Pseudomonas aeruginosa* is an opportunistic pathogen that causes a wide range of acute and chronic infections and is frequently associated with healthcare-associated infections. Because of its ability to rapidly acquire resistance to antibiotics, *P. aeruginosa* infections are difficult to treat. Alternative strategies, such as a vaccine, are needed to prevent infections*.* We collected a total of 413 *P. aeruginosa* isolates from the blood and cerebrospinal fluid of patients from 10 countries located on 4 continents during 2005–2017 and characterized these isolates to inform vaccine development efforts. We determined the diversity and distribution of O antigen and flagellin types and antibiotic susceptibility of the invasive *P. aeruginosa*. We used an antibody-based agglutination assay and PCR for O antigen typing and PCR for flagellin typing. We determined antibiotic susceptibility using the Kirby-Bauer disk diffusion method.

**Results:**

Of the 413 isolates, 314 (95%) were typed by an antibody-based agglutination assay or PCR (*n* = 99). Among the 20 serotypes of *P. aeruginosa*, the most common serotypes were O1, O2, O3, O4, O5, O6, O8, O9, O10 and O11; a vaccine that targets these 10 serotypes would confer protection against more than 80% of invasive *P. aeruginosa* infections. The most common flagellin type among 386 isolates was FlaB (41%). Resistance to aztreonam (56%) was most common, followed by levofloxacin (42%). We also found that 22% of strains were non-susceptible to meropenem and piperacillin-tazobactam. Ninety-nine (27%) of our collected isolates were resistant to multiple antibiotics. Isolates with FlaA2 flagellin were more commonly multidrug resistant (*p* = 0.04).

**Conclusions:**

Vaccines targeting common O antigens and two flagellin antigens, FlaB and FlaA2, would offer an excellent strategy to prevent *P. aeruginosa* invasive infections.

**Supplementary Information:**

The online version contains supplementary material available at 10.1186/s12866-021-02427-4.

## Background

*Pseudomonas aeruginosa* is an opportunistic human pathogen that causes acute life-threatening infections in elderly, critically ill and immunocompromised patients worldwide [1]. *P. aeruginosa* is a common cause of healthcare-associated infections (HAIs). Magill et al. [2] reported a point prevalence study in the United States which determined that *P. aeruginosa* was responsible for 7.1% of HAIs. This finding is supported by recent data from the National Healthcare Safety Network (NHSN) from 2015 to 2017 which reported *P. aeruginosa* as the fourth (8%) most frequently isolated pathogen in all HAIs in the U.S. [3]. Likewise, in the European Union, Walter et al. [4] performed a point prevalence study to investigate healthcare-associated pneumonia and determined that *P. aeruginosa* was the most commonly isolated pathogen (17%). This bacterium also causes chronic pulmonary infections in chronic wounds and cystic fibrosis (CF) patients [5, 6].

Severe life-threatening infections caused by *P. aeruginosa* are difficult to treat because of the organism’s limited susceptibility to antimicrobial agents [7]. Multi-drug resistant (MDR) *P. aeruginosa* is a serious concern for hospitalized patients. The SENTRY antimicrobial surveillance program, which collects data from 400 medical centers worldwide, showed that most of the *P. aeruginosa* isolates were from patients with pneumonia followed by bloodstream infections during 1997–2016 [8]. The SENTRY surveillance program also reported that 27.7 and 23.7% of *P. aeruginosa* from pneumonia and bloodstream infections, respectively, were MDR [8]. MDR isolates were most frequently isolated from Latin America followed by Europe, North America and Asia-Pacific. Unfortunately, no sites from Africa are included in the SENTRY program. The emergence of antibiotic resistance significantly limits therapeutic options for this bacterium.

O-polysaccharide (OPS), the most variable region of lipopolysaccharide (LPS), plays an important role in virulence and is responsible for conferring serogroup specificity [9]. Several serotyping systems have been proposed by various investigators; however, the most common serotyping scheme is the International Antigenic Typing Scheme (IATS). According to the IATS, *P. aeruginosa* have been classified into 20 different serotypes (O1 to O20) based on the structure of their O-polysaccharide [10, 11].

Studies have shown that isolates belonging to serotypes O1, O6, O11, and O12 account for > 65% of the *P. aeruginosa* infections [12, 13] and serotype O4 and O12 isolates are more frequently associated with resistance to various classes of antibiotics [14, 15]. Serotyping data indicates that *P. aeruginosa* isolates of serotypes O5, O6 and O11 are prevalent in burn wound infections [16] and O6 and O11 are the most prevalent serotypes in pneumonia [17, 18]. A study conducted at a Canadian hospital in 1992–1993 has found that O1, O3, O5, O6, O10, and O11 were the most frequently identified serotypes isolated from pus, urine, sputa and other sources [19]. Recently, in silico serotyping of > 1000 genomes of *P. aeruginosa* strains using whole genome sequencing revealed that 70% of the isolates were O3, O6, O11 or O12 [20]. Donta et al. [21] reported that serotypes O1, O2/O5, O3, O4, O6, O7, O10 and O16, represent 90% of bacteremic *P. aeruginosa* strains. Data describing the distribution of O serotypes among invasive *P. aeruginosa* from diverse geographical locations is lacking.

A study conducted by Lu et al. [17] showed a relationship between clinical outcome of pneumonia and *P. aeruginosa* serotypes. Serotype O1 was associated with mortality and serotypes O6 and O11 were prevalent in critically ill patients [17]. Previous studies also showed that some serotypes are more virulent than others; for instance, serotype O11 clinical isolates were found to secrete exotoxin U (ExoU), a toxin of the Type III Secretion System, more frequently than other serotypes [18] and in a murine model of pneumonia, serotype O11 was associated with increased lung injury [22].

The flagellum of *P. aeruginosa* consists of a polymer of flagellin (Fla) protein encoded by the *fliC* gene of serotype A (including sub-type A1 and A2) or B. There is no evidence of switching between serotype A and type B flagellin for any particular *P. aeruginosa* strain [23]. Antibodies to the flagellin of *P. aeruginosa* are protective which has been documented in pre-clinical models of subcutaneous infections, burn wounds and pneumonia [24]. It is not known whether there is a relationship between strains of particular flagellin types and antimicrobial susceptibility.

To control the spread of *P. aeruginosa* infection, an effective vaccine or other alternative immunotherapy is urgently needed. Despite decades-long efforts by the scientific community, no vaccine has yet been licensed to prevent infections by this pathogen [25]. Several groups have developed vaccines to prevent *P. aeruginosa* infections by targeting lipopolysaccharide (LPS), flagella, alginate, outer membrane proteins (OMPs) and proteins of the Type III Secretion System (T3SS) [26–31]. A double-blind randomized Phase 3 trial in cystic fibrosis patients with a bivalent *P. aeruginosa* A/B flagellin vaccine revealed robust and durable antibody titers, and modest but statistically significant protection against *P. aeruginosa* infection in cystic fibrosis patients [32]. Unfortunately, the company that manufactured this vaccine stopped production and it is not currently available, but these data establish the proof of concept that flagellin-based vaccines are efficacious. High molecular weight OPS have been used to develop a polysaccharide-based vaccine against *P. aeruginosa* [33]. We have extended this approach and created a vaccine using the novel Multiple Antigen Presenting System (MAPS) to target 8 *P. aeruginosa* O-serotypes (O1, O2, O3, O4, O5, O6, O10 and O11) [34].

In this study, we examined the diversity of O-antigen serotypes and flagellin types among invasive *P. aeruginosa* isolates to get better insight into globally circulating serotypes and their antibiotic susceptibility. The findings from the present study provide information about the global serotype distribution of this pathogen which will facilitate development of an effective vaccine against *P. aeruginosa*.

## Results

### Antibiotic resistance

In this study, we collected 413 invasive clinical isolates of *P. aeruginosa* from patients’ blood and cerebrospinal fluid from 10 countries (Table 1). Antibiotic susceptibility was determined for 370 *P. aeruginosa* invasive isolates by the disk diffusion method. Isolates obtained from Greece were excluded from all analyses involved in antimicrobial resistance because they were part of an MDR collection (Supplementary Fig. S1 and Fig. S2). Invasive *P. aeruginosa* strains were most frequently resistant to the antibiotic aztreonam (56%), followed by levofloxacin 42% (Table 2). We found 22% of strains were non-susceptible to carbapenems. The distribution of MDR *P. aeruginosa* varied by country. No MDR *P. aeruginosa* isolates were identified in Mali.

**Table 1:** Source and origin of *Pseudomonas aeruginosa* isolates used in this study

Region	Country	Institute of isolation	Source	Years of isolation	No. of strains
North America	USA	University of Maryland, Medical Center	Blood	2010 to 2015	116
		Duke University Medical Center	Blood	2012 to 2015	51
Europe	Sweden	Karolinska University Hospital	Blood	2015	25
	Greece	AHEPA University Hospital	Blood	2012 to 2016	43
	Denmark	Statens Serum Institute	Blood	2017	35
Africa	South Africa	NICD^a^ South Africa	Blood	2015	50
	Mali	CVD^b^-Mali	Blood/CSF	2005 to 2012	24
	DR Congo	ITM^c^ Belgium	Blood	2015	3
Asia	Japan	Nagoya University Hospital	Blood	2010 and 2015	14
	Singapore	Singapore General Hospital	Blood	2012 to 2016	25
	Pakistan	Aga Khan University	Blood	2015 to 2017	27

^a^National Institute for Communicable Diseases

^b^Center for Vaccine Development and Global Health

^c^Institute of Tropical Medicine, Antwerp

**Table 2:** Antibiotic resistance of invasive *P. aeruginosa*

Country^a^	No. of isolates	No. of isolates (%) non-susceptible to							MDR
		Aminoglycoside		Carbapenem	Cephalosporin	Fluoroquinolone	β-lactamase inhibitor	Monobactam	
		Amikacin	Gentamicin	Meropenem	Cefepime	Levofloxacin	Piperacillin-tazobactam	Aztreonam	
USA^b^	116	0 (0)	10 (8.6)	23 (19.8)	10 (8.6)	48 (41.4)	24 (21.0)	56 (48.3)	28 (24.1)
USA^c^	51	0 (0)	7 (14.0)	9 (17.6)	5 (10.0)	21 (41.2)	13 (25.5)	27 (53.0)	14 (27.4)
Denmark	35	2 (5.7)	2 (5.7)	6 (17.1)	5 (14.3)	20 (57.1)	5 (14.3)	27 (77.1)	8 (22.8)
Sweden	25	2 (8.0)	2 (8.0)	4 (16.0)	2 (8.0)	9 (36.0)	3 (12.0)	6 (24.0)	5 (20.0)
South Africa	50	8 (16.0)	15 (30.0)	18 (36.0)	17 (34.0)	25 (50.0)	15 (30.0)	42 (84.0)	20 (40.0)
Mali	24	1 (4.2)	1 (4.2)	0 (0)	0 (0)	1 (4.2)	0 (0)	8 (33.3)	0 (0)
DR Congo	3	0 (0)	2 (66.7)	1 (33.3)	1 (33.3)	2 (66.7)	2 (66.7)	2 (66.7)	2 (66.7)
Japan	14	0 (0)	0 (0)	4 (28.6)	0 (0)	4 (28.6)	0 (0)	7 (50.0)	2 (14.3)
Singapore	25	2 (8.0)	7 (28.0)	9 (36.0)	9 (36.0)	12 (48.0)	10 (40.0)	16 (64.0)	11 (44.0)
Pakistan	27	6 (22.2)	6 (22.2)	8 (29.6)	8 (29.6)	14 (51.8)	9 (33.3)	15 (55.5)	9 (33.3)
Overall	370	21 (5.7)	52 (14.0)	82 (22.2)	57 (15.4)	156 (42.2)	81 (22.0)	206 (55.7)	99 (26.7)

^a^Isolates collected from Greece were excluded

^b^Isolates were obtained from University of Maryland Medical Center

^c^Isolates were obtained from Duke University Medical Center

### Serotyping and global distribution of invasive *P. aeruginosa*

**Fig. 1:**
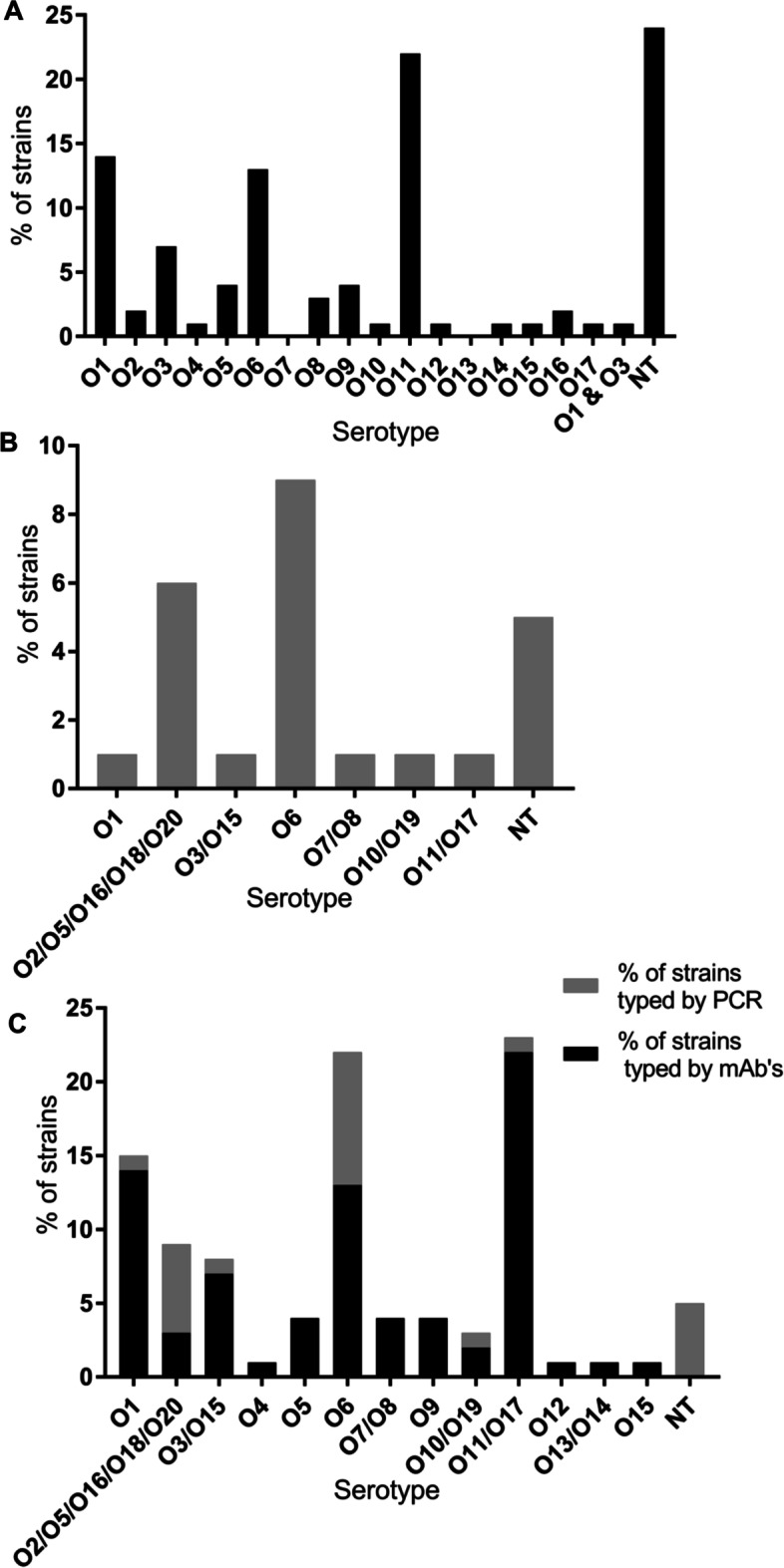
Distribution of O serotypes in 413 invasive *P. aeruginosa* isolates. **A** O serotypes were determined by slide agglutination assay. **B** Isolates that were non-typable (NT) by the slide agglutination assay were O-typed by PCR (*n* = 99). **C** Distribution of O serotypes using slide agglutination and PCR assays for O-antigen typing. mAb’s, monoclonal antibodies

All 413 *P. aeruginosa* invasive isolates were serotyped using a commercially available antibody-based serotyping kit (Supplementary Fig. S1). The distribution of serotypes is shown in Fig. 1A. The most prevalent serotypes were O11 (*n* = 89; 22%), O1 (*n* = 58; 14%) and O6 (*n* = 53; 13%) which accounted for around 50% of the *P. aeruginosa* strains that are circulating worldwide. Ninety-nine isolates (24%) were non-typable using the commercially available antibody-based serotyping kit. To serotype these non-typable isolates (*n* = 99), we used an O-antigen specific PCR (Fig. 1B). Unlike the antibody-based agglutination assay which can serotype *P. aeruginosa* strains into 17 O types, PCR can categorize isolates into 11 groups of gene clusters that are highly divergent from one another at the DNA sequence level [35]. The most prevalent serotype identified using PCR was O6 followed by the O2/O5/O16/O18/O20 cluster which comprised 9 and 6% of the isolates, respectively. We were able to serotype 95% (*n* = 393) of the *P. aeruginosa* isolates using both antibody-based agglutination and PCR methods. Only 5% (*n* = 20) of the isolates remained non-typable after employing both serotyping methods. We randomly selected 8 isolates that did not agglutinate with typing antibodies but were identified as O1, O2/O5/O16/O18/O20, O3/O15 or O6 strains by PCR and showed that they were able to produce O-side chains of the LPS (Supplementary Fig. S3). We have determined the serotype of 95% of the *P. aeruginosa* isolates using agglutination and PCR methods (Fig. 1C). Taken together, we found that the most common O serotypes amongst 413 strains include O1, O2/O5/O16/O18/O20, O3/O15, O6, O7/O8, O9, O10/O19 and O11/O17 which represented 92% of the isolates.

**Fig. 2:**
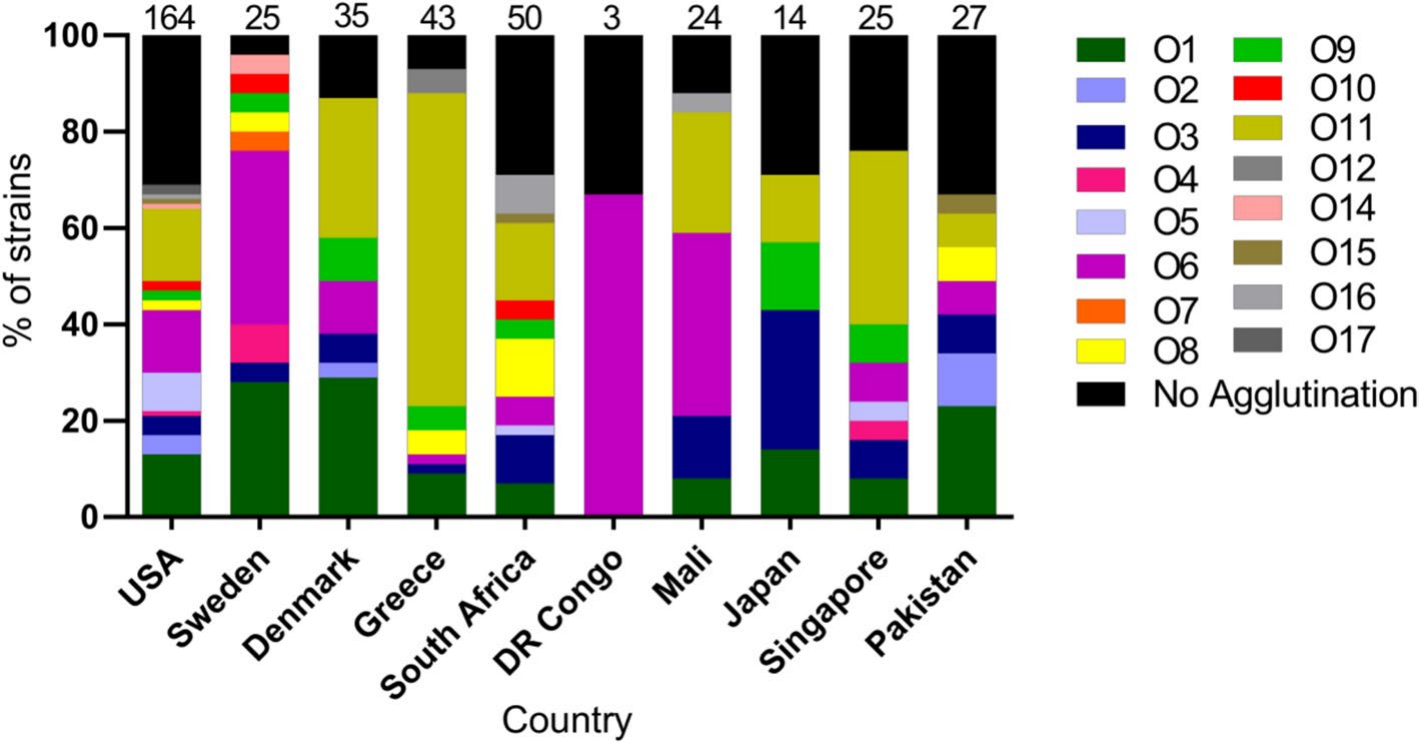
Diversity of *P. aeruginosa* O serotypes by country using an antibody based serotyping kit. The numbers on top of the graph represent the number of isolates tested from each country

We also determined the global serotype distribution of *P. aeruginosa* isolates among different countries using the agglutination data (Fig. 2). Overall, we observed diverse serotypes of invasive *P. aeruginosa* circulating worldwide. Clinically important O1, O6 and O11 serotypes were generally the most common in each country.

### Flagellin typing of *P. aeruginosa*

**Fig. 3:**
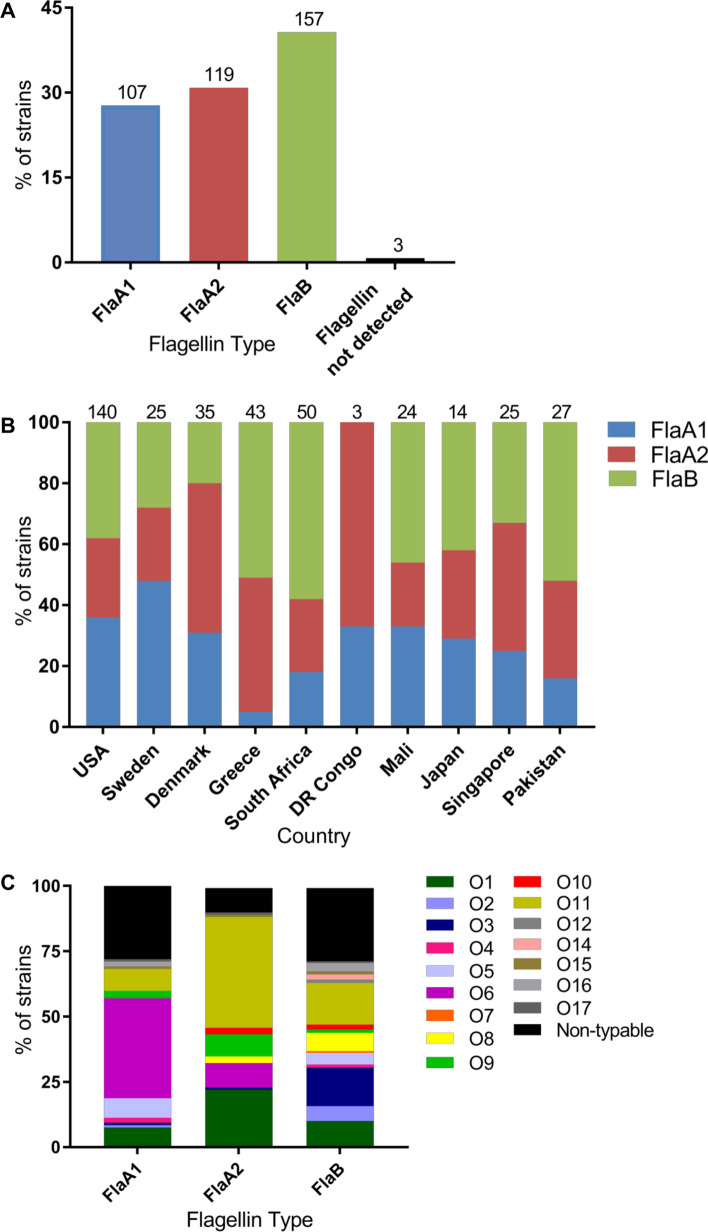
Distribution of flagellin types. **A** Distribution of flagellin types amongst 386 invasive *P. aeruginosa* isolates by PCR. The numbers on top of the graph represent the number of isolates of each flagellin type. **B** Distribution of flagellin types by country. The numbers on top of the graph represent the number of isolates tested from each country. **C** Distribution of O serotypes by flagellin type

The most common flagellin type among 386 (flagellin typing was not performed on 27 isolates due to logistic constraints) invasive *P. aeruginosa* isolates was FlaB (41%), followed by FlaA2 (31%) and FlaA1 (28%) (Fig. 3A). No PCR product was observed for three strains (one from Singapore which was not motile and two from Pakistan which were motile). We found that FlaB was prevalent in isolates from South Africa (58%), Pakistan (52%) and Greece (51%) whereas isolates from Denmark and Sweden were predominantly FlaA2 (49%) and FlaA1 (48%) (Fig. 3B). Overall, we observed diverse profiles of flagellin types among different countries. We observed a significant association between O serotypes and flagellin type (Chi-squared test, *p* < 0.001) where strains that possess FlaA1 were likely to be associated with serotype O6 (38.3%) and FlaA2 isolates were likely to be serotype O11 (42.4%) or O1 (22.9%) (Fig. 3C). Isolates with FlaB were likely to be associated with serotype O11 (15.9%) and O3 (14.6%). Furthermore, non-typable O serotypes were associated with FlaA1 and FlaB.

**Fig. 4:**
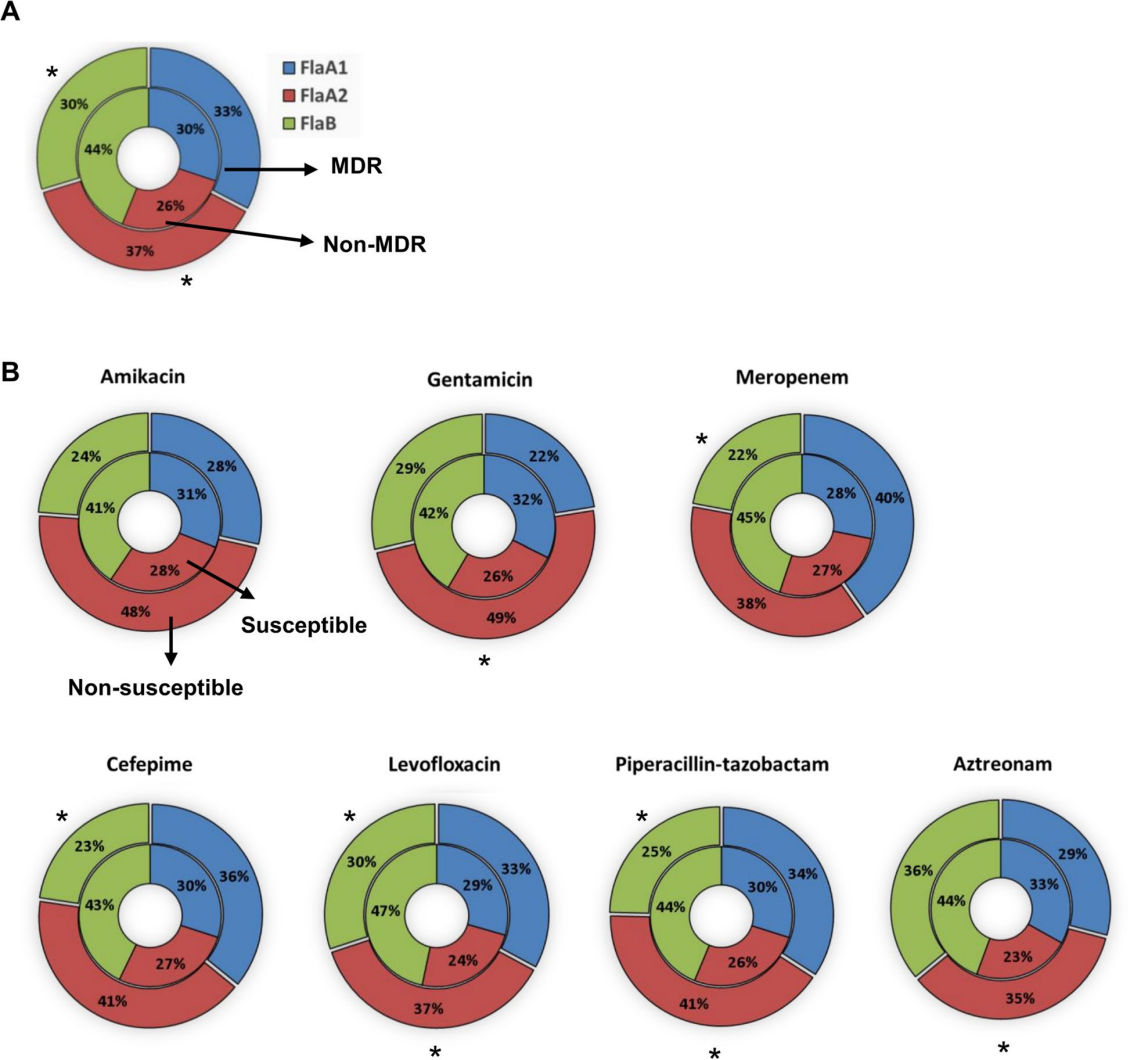
Association between flagellin types and antibiotic resistance in clinical *P. aeruginosa* strains. **A** Inner circle of the pie represents the percentage of flagellin types that are non-MDR and the outer circle of the pie represents the percentage of flagellin types that are MDR. **B** The percentage of flagellin types in susceptible isolates (inner circle) and in non-susceptible isolates (outer circle) to each antibiotic. Statistically significant differences between MDR and non-MDR and susceptible and non-susceptible groups are indicated by asterisks (*). Isolates obtained from Greece were excluded from the analysis

To determine if particular flagellar types were associated with antibiotic resistance, we evaluated the distribution of flagellar types among susceptible and non-susceptible strains for each antibiotic and for MDR and non-MDR strains. We observed that FlaA2 was significantly (*p* < 0.05) associated with MDR. In contrast, FlaB was more common amongst non-MDR than MDR strains (*p* < 0.05) (Fig. 4A). The percentage of flagellar types in susceptible and non-susceptible groups to each antibiotic is shown in Fig. 4B. We found significantly more FlaA2 isolates that were non-susceptible to gentamicin, levofloxacin, piperacillin-tazobactam and aztreonam compared to susceptible (*p* < 0.05). In contrast, FlaB type was significantly more common in isolates susceptible to meropenem, cefepime, levofloxacin and piperacillin-tazobactam. We did not find any significant difference in FlaA1 distribution in susceptible versus non-susceptible strains.

## Discussion

In this study, we collected more than 400 invasive *P. aeruginosa* isolates from geographically diverse locations to determine their serotype distribution, flagellin types and antibiotic susceptibility pattern to understand the epidemiology of this pathogen and to guide vaccine development efforts. The challenges for developing an effective vaccine against *P. aeruginosa* are multifactorial, interplaying between the pathogen’s virulence factors, ability to utilize multiple pathways to cause infection, antibiotic resistance, emerging high-risk clones and host factors [36]. Emergence of MDR *P. aeruginosa* is a serious public health concern.

We observed that 26.7% of the *P. aeruginosa* isolates tested (*N* = 370) were MDR. Our data are similar to those of the SENTRY surveillance program which found 23.7% MDR out of 52,022 *P. aeruginosa* isolates collected in 1997–2016 from > 400 medical centers representing Asia-Pacific, Europe, Latin America and North America [8]. We found the highest prevalence of MDR isolates from Singapore, South Africa and Democratic Republic of the Congo (albeit only 3 isolates of the latter were tested).

Cefepime is a widely used fourth generation cephalosporin that has potent antimicrobial activity against *Pseudomonas* [37]. However, resistance of *P. aeruginosa* to cefepime has been observed in several other studies [37–39] as well as in this study. This observation of resistance to cefepime is of concern as Akhabue et al. [38] showed a significant association between infections caused by invasive cefepime-resistant *P. aeruginosa* and increased mortality among hospitalized patients.

In our study, 22% of the *P. aeruginosa* were resistant to meropenem, an antibiotic that has been considered as the most effective agent for the treatment of *P. aeruginosa* infections [40]. Our observations are similar to that of the SENTRY surveillance program which reported that 22.3% of bloodstream isolates were resistant to meropenem [8]. In contrast, other studies have reported higher resistance to carbapenems; among 2070 *P. aeruginosa* isolates from 27 hospitals located in 14 European and Mediterranean countries, 94.5% were resistant to imipenem, 81.6% to meropenem and 57.3% to doripenem using Clinical and Laboratory Standards Institute (CLSI) breakpoints [41]. The increased prevalence of carbapenem resistance among the 2070 *P. aeruginosa* isolates reported by Castanheira et al. might be due to the varied source of the isolates which were obtained from bloodstream, nosocomial respiratory tract infections, skin, urine and other unknown sources (in contrast to our study where isolates were obtained from blood or cerebrospinal fluid). It is worth noting that the World Health Organization (WHO) recently ranked carbapenem-resistant *P. aeruginosa* as critical in their global priority pathogens list of MDR bacteria to help in prioritizing the research and development of new and effective antibiotic treatments [42].

One of the objectives of this study was to identify the most common serotypes of *P. aeruginosa* circulating to inform development of O-polysaccharide-based vaccines. We found that the distribution of serotypes varied amongst investigation sites and countries, but the most prevalent serotypes were O1, O6 and O11, which comprise about 50% of tested isolates. These results are concordant with findings from previous studies [13]. To our knowledge, this is the first study to evaluate the distribution of *P. aeruginosa* serotypes in invasive infections from multiple (ten) countries with diverse geographical locations. Our antibody-based serotyping data indicated that > 70% of the isolates belonged to 10 serotypes (O1, O2, O3, O4, O5, O6, O8, O9, O10 and O11). However, antibody-based serotyping data in combination with the molecular method, determined that > 80% of the strains belonged to these 10 serotypes. Thus, targeting these 10 serotypes in a vaccine could conceivably confer protection against infection caused by a large number of invasive *P. aeruginosa* strains. We and others have observed a low prevalence of serotype O12 in clinical strains [13, 18]; however, other studies have shown that isolates of serotype O12 are more frequently associated with antibiotic resistance [14]. Therefore, serotype O12 might be included in future vaccine programs to prevent spread of antimicrobial resistant strains.

In the current study, the most common flagellin type was FlaB (40.6%) followed by FlaA2 (30.8%) and FlaA1 (27.7%) and distribution of flagellin types among countries was diverse. We found that strains with FlaA1 were significantly associated with O6 and O11; FlaA2 strains were frequently serotyped as O1 or O11; and isolates that possessed FlaB were significantly associated with O11 and O3. We also observed that FlaA2 was the predominant flagellin type among MDR *P. aeruginosa* strains and was associated with resistance to gentamicin, levofloxacin, piperacillin-tazobactam and aztreonam. The role of flagella or flagellin proteins in virulence of *P. aeruginosa* have been previously documented [43]; however, to our knowledge, no association between flagellin type and antibiotic resistance has been described for *P. aeruginosa*. Given the conserved nature and limited flagellin types in *P. aeruginosa*, it would be worthwhile to include flagellin proteins as a vaccine component to improve vaccine coverage. Our data suggests that although FlaB was the most common flagellin type, it would also be important to target FlaA2 strains which are associated with antibiotic resistance.

## Conclusions

We determined antibiotic susceptibility of invasive *P. aeruginosa* collected from 10 countries located on 4 different continents. The distribution of MDR *P. aeruginosa* varied by country. *P. aeruginosa* strains were most frequently resistant to the antibiotic aztreonam, followed by levofloxacin and 22% of the invasive isolates were non-susceptible to carbapenems. Our data suggest that a multivalent vaccine that targets 10 O serotypes (O1, O2, O3, O4, O5, O6, O8, O9, O10 and O11) would confer protection against > 80% of circulating invasive *P. aeruginosa* isolates. We also determined that if one was to include flagellin antigens in a vaccine, it would be important to include FlaB (most common flagellin type) and FlaA2 (associated with antibiotic resistance). The findings from this study will help to guide efforts to develop an effective vaccine against invasive *P. aeruginosa.*

## Materials and methods

### Bacterial isolates collection and growth conditions

Four hundred and thirteen invasive clinical isolates of *P. aeruginosa* were collected during 2005–2017 from ten countries located in four different geographic regions (Table 1). Bacterial isolates were collected from patients’ blood and cerebrospinal fluid. As reference strains, the International Antigenic Typing Scheme (IATS) serotype-type specific strains, IATS O1, O2, O3, O4, O5, O6, O7, O8, O9, O10, O11, O12, O13, O14, O15, O16, O17, were used. *P. aeruginosa* was isolated and identified at each site using standard microbiologic protocols. As needed, identity was confirmed by morphological characteristics and biochemical profile using API 20NE (bioMérieux, Durham, NC) at the Center for Vaccine Development and Global Health (CVD), University of Maryland, Baltimore, Maryland. All confirmed *P. aeruginosa* isolates were preserved in Tryptic Soy Broth (TSB) supplemented with 15% (vol/vol) glycerol and stored at − 80 °C for future use. When needed, bacteria were streaked for single colonies on animal product-free Hy-Soy agar plates (0.5% sodium chloride, 1% animal free soytone [TEKNova, Hollister, CA], 0.5% Hy-Yest 444 [Kerry Biosciences, Beloit, WI]). For routine culture, cells were incubated at 37 °C for 18–24 h unless otherwise indicated. For motility tests, bacteria were stab inoculated using a sterile pipette tip onto a motility medium containing 1% tryptone, 0.5% NaCl, and 0.4% agar and the plates were incubated for 18 h at 37 °C.

### Antibiotic susceptibility testing

Antibiotic susceptibility studies were performed by Kirby-Bauer disk diffusion method according to CLSI guidelines (M100-S29). The following antibiotics were tested in this study: amikacin (30 μg), aztreonam (30 μg), cefepime (30 μg), gentamicin (10 μg), meropenem (10 μg), piperacillin-tazobactam (110 μg) and levofloxacin (5 μg). Cartridges of antibiotic impregnated discs were purchased from Becton Dickinson Company Ltd. (Franklin Lakes, NJ). Antibiotic susceptibility was determined by measuring the diameter of the zone of inhibition and the results were interpreted as susceptible (S), intermediate (I) and resistant (R) based on CLSI guidelines [44]. The criterion used in this study for defining multidrug-resistant (MDR) in *P. aeruginosa* was defined as non-susceptibility (including both resistant and intermediate) to three or more classes of antibiotics [45]. In this study, non-MDR isolates were defined as those susceptible to all antibiotics or resistant to 1 or 2 classes of antibiotics.

### O-antigen serotyping

*P. aeruginosa* isolates were serotyped by slide agglutination using commercially available antibodies specific to *P. aeruginosa* (MediMabs, Montreal, Canada) according to the manufacturer’s instructions. Briefly, *P. aeruginosa* was streaked onto Hy-Soy agar medium and incubated for 16–18 h at 37 °C. After incubation, cells were serotyped using three groups of pooled antisera (pool A, pool B and pool C) followed by a panel of 17 monovalent antisera (O1, O2, O3, O4, O5, O6, O7, O8, O9, O10, O11, O12, O13, O14, O15, O16 and O17) based on the IATS serotyping system. Cells producing clear aggregation and clumping in the presence of antibody were considered as serotype-positive, whereas cells that remained uniformly suspended were considered as serotype-negative.

Isolates which were non-typable by slide agglutination assay were O-serotyped by PCR according to a method described previously [35]. Genomic DNA was extracted from each of the non-typable isolates using the GenElute genomic DNA purification kit (Sigma-Aldrich, Billerica, MA) according to the manufacturer’s instructions. O-antigen specific PCR was performed as follows: each 25 μl reaction mixture contained 2.5 μl of 10× Greentaq buffer (Genscript, Piscataway, NJ), 0.5 μl of 10 μM deoxynucleotide triphosphate mix (dNTPs) (Genscript), 1.0 μl of 10 μM forward and reverse primer mix (See Table S1 for details of primers), 0.5 U of Greentaq DNA polymerase (Genscript) and 40 ng of DNA template. Cycling parameters were 95 °C for 3 min followed by 35 cycles of 95 °C for 30 s, 60 °C for 30 s and 72 °C for 45 s to 1.5 min (depending on the primer mixes) with a final extension at 72 °C for 5 min. PCR amplicons (10 μl) were electrophoresed on 1.0% agarose gels and photographed using a ChemiDoc™ MP imaging system (Bio-Rad, Hercules, CA). O-serotype was determined based on the presence or absence of an amplicon.

### Lipopolysaccharide (LPS) extraction

Eight representative non-typable *P. aeruginosa* isolates were selected to confirm expression of O polysaccharide. Crude LPS was extracted from *P. aeruginosa* isolates, electrophoresed and visualized by staining. *P. aeruginosa* strain PAK was used as a positive control. Cells were grown overnight in 3 ml of Hy-Soy broth in a shaking incubator at 37 °C and 200 rpm. After incubation, cells were pelleted by centrifugation followed by washing the pellet twice with 1 × PBS to remove any pyocyanin from the media (to remove pigments that can affect spectrophotometric measurements). Pellets were resuspended in PBS and optical density (OD) at 600 nm was adjusted to 1.0. One milliliter of the OD_600_ standardized culture was centrifuged at 15,600×*g* for 1 min. Supernatant was discarded carefully without disturbing the pellet. The pellet was suspended in 250 μl of Laemmli buffer 1× with β-mercaptoethanol (BME). Samples were boiled at 100 °C for 20 min, allowed to cool at room temperature and then 20 μg of proteinase K (Qiagen Germantown, MD) was added to the solution. The solution was incubated at 55 °C for 4 h. Proteinase K was then heat inactivated at 100 °C for 10 min. For electrophoresis, 25 μl of the sample and 2 μl of CandyCane glycoprotein molecular weight standard (Invitrogen Waltham, MA) was loaded onto a NuPAGE 4–12% Bis-Tris Gel (Invitrogen Carlsbad, CA) and LPS visualized using the Pro-Q® Emerald 300 lipopolysaccharide gel stain kit according to the manufacturer’s instructions (Thermo Fisher Waltham, MA).

### Flagellin typing of *Pseudomonas aeruginosa*

To determine flagellin types for *P. aeruginosa* isolates, the central region of the *fliC* gene was amplified by PCR and sequenced using primer pair CW46_F (5′-GGCCTGCAGATCNCCAA-3′) and CW45_R (5′-GGCAGCTGGTTNGCCTG-3′) [46]. The primer pair used in this study produced amplicons that can differentiate between A and B type flagellin. Boiled lysates of *P. aeruginosa* isolates were used as DNA template for performing flagellin-specific PCR. Four to six colonies were suspended in 150 μl of nuclease free water. Samples were boiled at 100 °C for 10 min and then pelleted by centrifugation at 14,000 rpm for 5 min. The supernatants were collected and used as DNA template. PCR was performed as follows: each 50 μl reaction mixture contained 5.0 μl of 10× Greentaq buffer (Genscript), 1.5 μl of 2 mM deoxynucleotide triphosphate mix (dNTPs) (Genscript), 3.0 μl of 10.0 μM forward and reverse primer mix, 1.0 U of Greentaq DNA polymerase (Genscript) and 1.0 μl of DNA template. Reactions were incubated at 95 °C for 3 min followed by 30 cycles of denaturation at 95 °C for 30 s, annealing at 56 °C for 45 s, and extension at 72 °C for 1.5 min, with a final extension at 72 °C for 5 min. The PCR products were analyzed by agarose gel electrophoresis. To differentiate between A1 and A2 subtypes, a new set of primers (PA_FLIC_F: 5′-ATGGCCTTGACCGTCAAC-3′ and PA_FLIC_R: 5′-GCGCAGCAGGCTCAGAAC-3′) were used to generate *fliC* amplicons and for sequencing. PCR was performed according to the conditions described above.

Amplicons were then purified using the Qiagen PCR purification kit (Germantown, MD) according to the manufacturer’s protocol. Purified PCR products were sequenced by Genewiz (South Plainfield, NJ). Sequences were de novo assembled and compared with reference sequences using the Geneious software package.

### Statistical analysis

The proportion of susceptible versus resistant strains for each flagellin type was analyzed by Chi-squared analysis with Yate’s correction using GraphPad Prism version 7 software (GraphPad Software, San Diego). The significance level was *p* < 0.05. The association between O serotype and flagellin type was determined by Chi-squared test using Stata/SE version 16 software.

## Supplementary Information

**Additional file 1: Table S1.** Primers used for O antigen serotyping. **Figure S1.** The number of *P. aeruginosa* isolates tested by each method. *Flagellin typing was not performed on 27 isolates from Duke University due to logistic constraints. **Figure S2.** Antibiotic resistance of isolates from Greece. **Figure S3.** The O-antigen profiles of representative non-typable *P. aeruginosa* isolates determined by SDS-PAGE. LPS from eight non-typable *P.aeruginosa* strains were extracted and separated on a 4–12% Bis-Tris SDS-polyacrylamide gel and stained with Pro-Q Emerald 300 according to manufacturer’s instructions. CandyCane glycoprotein molecular weight standard (ladder), *P. aeruginosa* strain PAK (+ve control).

## Data Availability

All data generated or analyzed during this study are included in this published article (and its supplementary information files). DNA sequences used to determine the flagellin type are available in GenBank (accession numbers OL354146 – OL354417).
