# Supplementary material for: Distribution of serotypes and antibiotic resistance of invasive Pseudomonas aeruginosa in a multi-country collection
Source: BMC Microbiol. 2022 Jan 6;22:13. doi: 10.1186/s12866-021-02427-4 (PMC8732956; doi:10.1186/s12866-021-02427-4)
Supplement: Supplementary file 1 — Additional file 1: Table S1. Primers used for O antigen serotyping. Figure S1. The number of P. aeruginosa isolates tested by each method. *Flagellin typing was not performed on 27 isolates from Duke University due to logistic constraints. Figure S2. Antibiotic resistance of isolates from Greece. Figure S3. The O-antigen profiles of representative non-typable P. aeruginosa isolates determined by SDS-PAGE. LPS from eight non-typable P.aeruginosa strains were extracted and separated on a 4–12% Bis-Tris SDS-polyacrylamide gel and stained with Pro-Q Emerald 300 according to manufacturer’s instructions. CandyCane glycoprotein molecular weight standard (ladder), P. aeruginosa strain PAK (+ve control). [file 12866_2021_2427_MOESM1_ESM.pdf]

Table S1. Primers used for O antigen serotyping.

	Primer Sequence	Product	Size (bp)
1A	TGCTCTTTGGCTTCTTGATTCTTG	Primer 1, O1-specific product	396
1B	TCTACACCGCCAGAACTACCTAGCT	Primer 2, O1-specific product	
2A	GCGGCTTTTATCAACCGTGTCGCA	Primer 1, O2, O5, O16, O18, and O20	1217
2B	TCCCTTGGCACACTGGAAGGACAT	Primer 2, O2, O5, O16, O18, and O20	
3A	GCGTCGTTGTTCAGTTTGGACGTG	Primer 1, O3 and O15	406
3B	TCTGGAAAACCTGAGCAGCCGTCC	Primer 2, O3 and O15	
4A	GCTAATAACGGAAGGACCTTGAAT	Primer 1, O4-specific product	551
4B	TAAAAGCTCGGCGTAACGCTTATG	Primer 2, O4-specific product	
5A	ATTGGCTAGTGCTACACGAGTGCA	Primer 1, O6-specific product	380
5B	CGAATTAGCTTGCTCTTCAGGAAAG	Primer 2, O6-specific product	
6A	GGAATGTCGCTCTCGTTTCAAGTG	Primer 1, O7 and O8	451
6B	CTAGTATTCATCAACTGCTGTAC	Primer 2, O7 and O8	
8A	CAGCAGGGAATATCGCTTGAACAGT	Primer 1, O10 and O19	547
8B	CTATAATGCATTAGCGACTCACCG	Primer 2, O10 and O19	
9A	TACTTCACCCATAGCTAGCGCTCTA	Primer 1, O11 and O17	452
9B	TTCTCTCTCAACTTAACCGTGGCC	Primer 2, O11 and O17	
10A	CTATTCAGTCGATGATCCTTGTG	Primer 1, O13 and O14	488
10B	AGCAGTTAGCAAATTTCACTCTCCAGCC	Primer 2, O13 and O14	

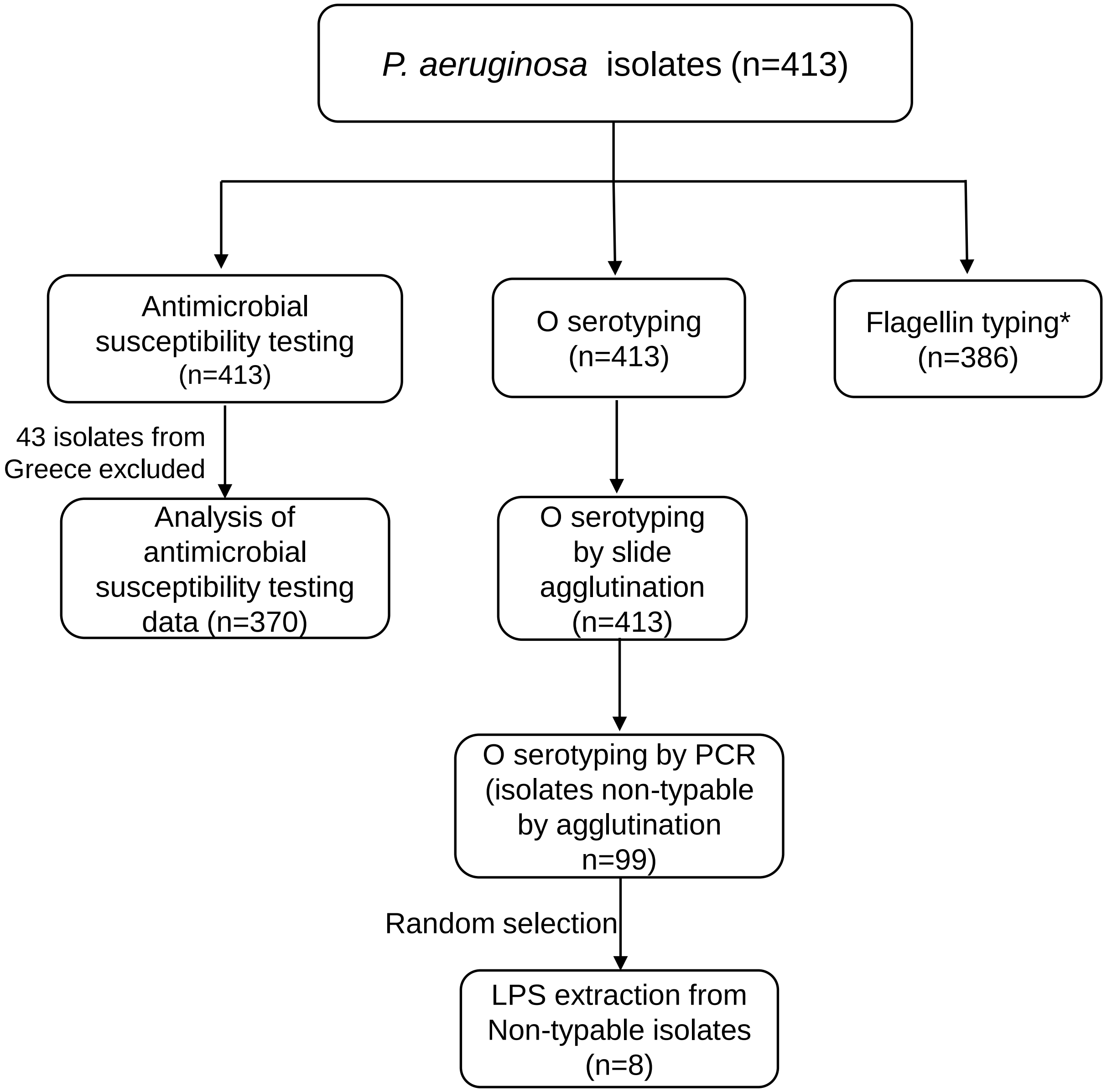

Figure S1. The number of *P. aeruginosa* isolates tested by each method. *Flagellin typing was not performed on 27 isolates from Duke University due to logistic constraints.

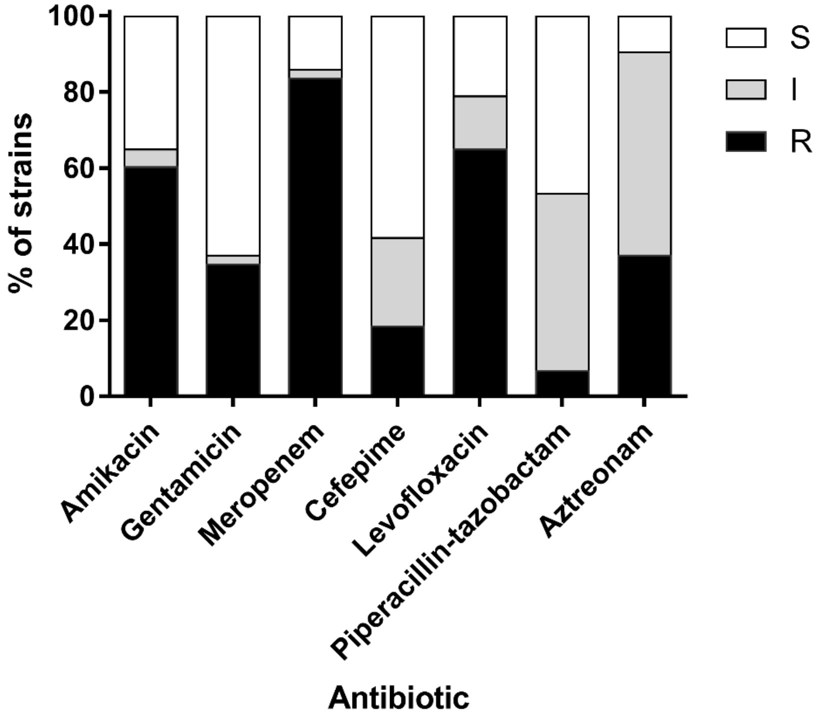

Figure S2. Antibiotic resistance of isolates from Greece.

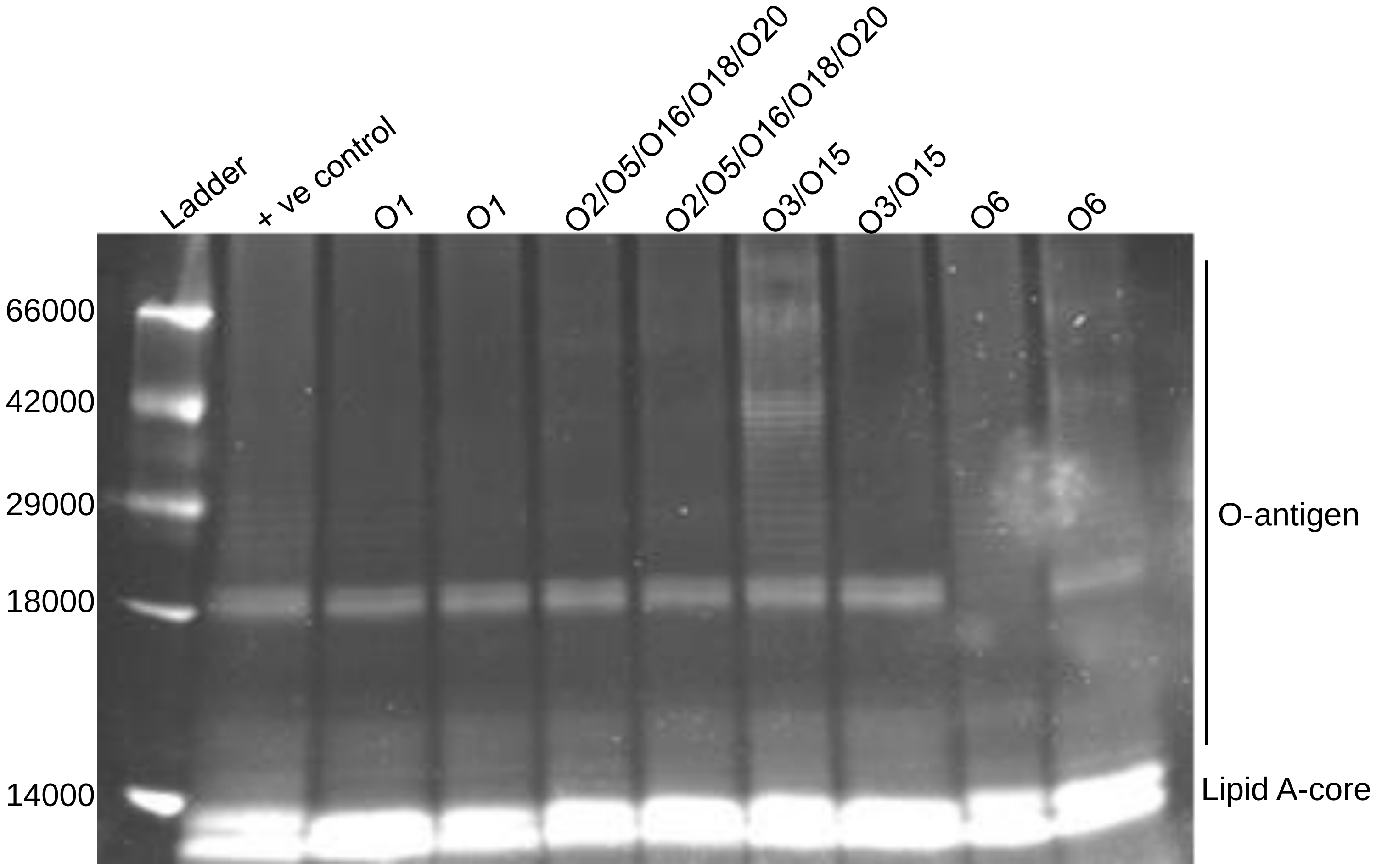

Figure S3. The O-antigen profiles of representative non-typable *P. aeruginosa* isolates determined by SDS-PAGE. LPS from eight non-typable *P. aeruginosa* strains were extracted and separated on a 4-12% Bis-Tris SDS-polyacrylamide gel and stained with Pro-Q Emerald 300 according to manufacturer's instructions. CandyCane glycoprotein molecular weight standard (ladder), *P. aeruginosa* strain PAK (+ve control).
